# New Pharmacological Tools to Target Leukocyte Trafficking in Lung Disease

**DOI:** 10.3389/fimmu.2021.704173

**Published:** 2021-07-21

**Authors:** Kylie B. R. Belchamber, Michael J. Hughes, Daniella A. Spittle, Eloise M. Walker, Elizabeth Sapey

**Affiliations:** ^1^ Birmingham Acute Care Research Group, Institute of Inflammation and Ageing, University of Birmingham, Birmingham, United Kingdom; ^2^ NIHR Clinical Research Facility Birmingham, University Hospitals Birmingham NHS Foundation Trust, Birmingham, United Kingdom

**Keywords:** neutrophil (PMN), respiratory, ageing, proteinase, chemotaxis, monocyte

## Abstract

Infection and inflammation of the lung results in the recruitment of non-resident immune cells, including neutrophils, eosinophils and monocytes. This swift response should ensure clearance of the threat and resolution of stimuli which drive inflammation. However, once the threat is subdued this influx of immune cells should be followed by clearance of recruited cells through apoptosis and subsequent efferocytosis, expectoration or retrograde migration back into the circulation. This cycle of cell recruitment, containment of threat and then clearance of immune cells and repair is held in exquisite balance to limit host damage. Advanced age is often associated with detrimental changes to the balance described above. Cellular functions are altered including a reduced ability to traffic accurately towards inflammation, a reduced ability to clear pathogens and sustained inflammation. These changes, seen with age, are heightened in lung disease, and most chronic and acute lung diseases are associated with an exaggerated influx of immune cells, such as neutrophils, to the airways as well as considerable inflammation. Indeed, across many lung diseases, pathogenesis and progression has been associated with the sustained presence of trafficking cells, with examples including chronic diseases such as Chronic Obstructive Pulmonary Disease and Idiopathic Pulmonary Fibrosis and acute infections such as Pneumonia and Pneumonitis. In these instances, there is evidence that dysfunctional and sustained recruitment of cells to the airways not only increases host damage but impairs the hosts ability to effectively respond to microbial invasion. Targeting leukocyte migration in these instances, to normalise cellular responses, has therapeutic promise. In this review we discuss the current evidence to support the trafficking cell as an immunotherapeutic target in lung disease, and which potential mechanisms or pathways have shown promise in early drug trials, with a focus on the neutrophil, as the quintessential trafficking immune cell.

## Introduction

The lungs, especially the alveolar network, are the area of the body where the external environment is in closest proximity to the circulating blood. The average diameter of the alveolar membrane is 0.2µm, and each minute, approximately 5L of blood and 5-8L of air (and the pollutants and microbes contained therein) pass through these organs, which have an internal surface area of 50-75 square metres. The lungs serve to enable gaseous exchange, but also need to preserve health by preventing damage caused by infections or inflammation. In health, the lungs maintain homeostasis through complex interactions between the lung microbiome (defined recently as the characteristic microbial community occupying the lungs, prone to change in time and scale and thought crucial for host function and health) (1), resident immune cells and defences and the trafficking of non-resident immune cells from the systemic circulation to the lung in the presence of more challenging inflammation, infection or injury.

Non-resident immune cells include neutrophils, eosinophils and monocytes, all of which are involved in the inflammatory process. The exact make up of both the trafficking cell type and cellular phenotype within cell types depends on the nature of the challenge, but an optimal response includes a swift and accurate recruitment of cells to the location of the injury or infection, clearance of the threat (be that pathogens or inflamed/necrotic tissue) through phagocytosis, and then resolution of inflammation *via* programmed cell death and clearance by efferocytosis or expectoration (within sputum) or retrograde migration back into the circulation (2). Phagocytosis of pathogens should lead to pathogen-killing through exposure to proteinases (especially in the case of neutrophils), bactericidal proteins or reactive oxygen species, combined and contained within phagolysosomes. This intracellular process limits host tissue exposure to injurious enzymes, but extracellular release does occur (as part of degranulation, so called ‘sloppy eating’ or during NETosis) and here, local tissue damage is unavoidable, although limited by the presence of anti-oxidants and anti-proteinases (3).

Pro and anti-inflammatory signals leading to immune cell recruitment and immune cell clearance are held in exquisite balance by cross talk between resident tissue and the migratory cells as the inflammatory challenge is overcome. When these processes go awry, through excessive, sustained cell recruitment, inaccurate migration, or impaired clearance; unresolved inflammation can lead to lung damage and contribute to the development of chronic lung disease. This can lead to a vicious cycle of lung damage, described first in Cole’s theory of bronchiectasis [a suppurative lung disease (4)], where tissue damage leads to an increased susceptibility to infection, which leads to immune cell recruitment and degranulation, with proteinases capable of digesting all components of the extracellular matrix, which leads to increased inflammation and subsequent on-going tissue damage. There is significant interest in therapeutically breaking this cycle, potentially limiting subsequent lung damage and maintaining lung health.

**Figure 1:**
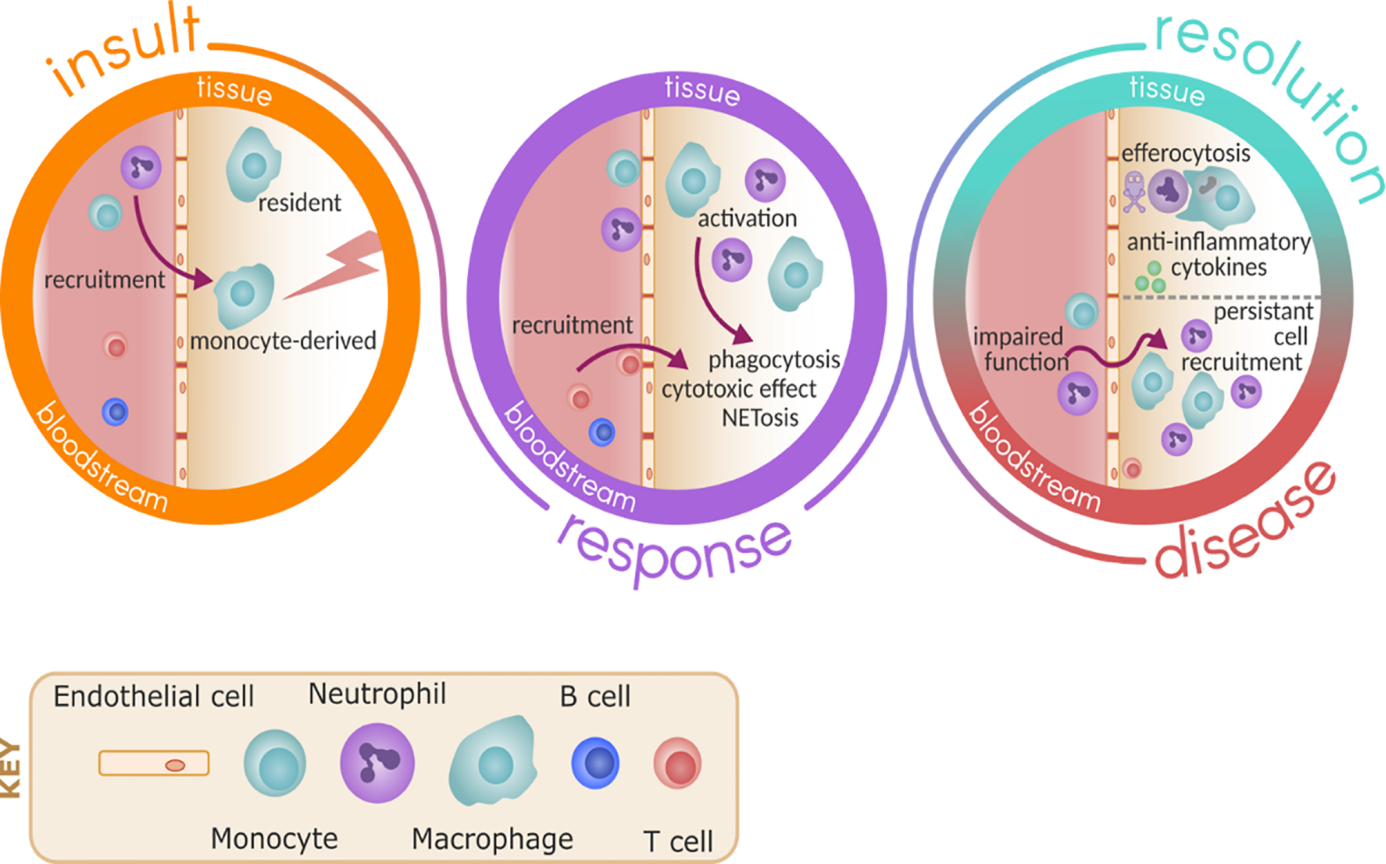
Immune response to inflammation and infection. Upon insult, either due to pathogen or sterile injury, resident immune cells such as macrophage are ready to respond and promote the recruitment of monocytes and neutrophils *via* activation of the endothelium. As part of the response, monocytes differentiate in the tissue to macrophage and these cells become activated to respond to the insult, promoting further recruitment of other immune cells such as T cells and carrying out effector functions including phagocytosis and NETosis. In health, resolution follows by death of neutrophils and clearance by efferocytosis, promoting the release of anti-inflammatory cytokines and repair. In disease, the persistent recruitment of immune cells and potential impaired effector functions of these cells perpetuate inflammation and damage.

Initially it was assumed that excessive immune cell recruitment to the lung was a normal, physiological response to a pathological stimulus. In this model, only the recruiting stimuli (the lung inflammation or the microbe) could be targeted to reduce cell infiltration. It was thought that targeting the trafficking immune cell would lead to immunoparesis and impair the ability to respond to subsequent infections, placing the host at risk. However, there is increasing evidence of altered and dysfunctional migrating cell behaviour in chronic and acute lung disease (5, 6), and emerging evidence that targeting leukocyte trafficking may improve these cells responses to infection while reducing absolute numbers of cells in the lungs, thus reducing the inflammatory burden. See **Figure 1** for an overview of this.

This review will discuss the current evidence to support the trafficking cell as an immunotherapeutic target in lung disease, and which potential mechanisms or pathways have shown promise in early drug trials, with a focus on the neutrophil, as the quintessential trafficking immune cell.

## Leukocyte Trafficking From the Blood

### Pro-Migratory Signals

Inflammation within the lung parenchyma leads to the release of a milieu of cytokines and chemokines from damaged epithelial cells, as well as activated alveolar macrophages and other resident or recruited immune cells such as neutrophils and T cells. Chemokines attract leukocytes with varying affinity and capacity. They are divided into groups based on the position of their conserved cysteine residues, with the CXC and CC families the most important for inflammatory disease (7). CXCL-8 and CXCL2 are important neutrophil chemokines, acting *via* tCXCR-1 and CXCR-2 receptors (8), alongside monocyte chemoattractant protein (MCP-1) for monocytes, which acts on the CCL2 receptor, resulting in monocyte recruitment and macrophage activation (9). These topics have been extensively reviewed elsewhere (10).

Initially it was thought that there was a simple relationship between the release of Damage-associated molecular patterns (DAMPs) and leukocyte recruitment. However, the complexity of signalling cascades from inflamed tissues is increasingly recognised. As vital components of the host defence, leukocytes must sense, prioritize and integrate all of the chemotactic cues from the environment into a migration response towards damaged tissues (11). To achieve this, neutrophils express more than 30 different receptors able to sense pro-inflammatory mediators and modulate neutrophil migration (12), whereas monocytes express various receptors depending on their subset (13).

The migration of neutrophils to inflamed tissues is thought to occur in phases. Early neutrophil recruitment (“scouting” cells) respond to tissue DAMPs through the SRC family kinase LYN. DAMPs induce the production of CXC-chemokines and leukotrienes from surrounding tissues (14). Early-arriving neutrophils add to this inflammatory cocktail, as, through activation, neutrophils directly and indirectly promote further secretion of CXCL8 and leukotriene B4 (LTB4) to induce further neutrophil recruitment from the circulation. The fine control of neutrophil extravasation is seen with CXCL1 promoting crawling of neutrophils along blood endothelial cells and then CXCL2 promoting unidirectional movement across the endothelium (15). In infection, the release of pathogen-associated molecular patterns (PAMPs) and the presence of other recruited immune cells prolongs and amplifies neutrophil infiltration. There appears to be a signal hierarchy with DAMPS and cytokines such as CXCL8 forming a migratory “start” signal which can be superseded or ignored in the presence of PAMPs such as fMLP (16, 17).

Monocytes exert many of their functions outside the vascular compartment, thus requiring trafficking to tissues. Monocytes in the tissues respond to chemokines and cytokines, differentiating into macrophages or dendritic cells during infection, as well as wound-associated macrophages or tumour induced myeloid suppressor cells (18). However, monocytes can also remain un-differentiated, at least in the resting state.

### The Components of Leukocyte Trafficking From the Systemic Circulation

In health, neutrophils and monocytes are released from the bone marrow in a quiescent state and maintain this in the circulation during homeostasis. They become primed in response to an initial activation signal *via* a plethora of agents, including bacterial products, cytokines and metabolic cues (19) and can then become activated whereby effector responses are deployed (20). Originally, the focus on neutrophil priming was on enhancing the ROS response (19), but is now known to also control other aspects of cell function including adhesion (21) and chemotaxis (22). Primed neutrophils also show a slower transit time through the lung vasculature, shown in patients that have inflammatory lung diseases such as COPD (23) or even low-grade inflammation (24). Priming of other immune cells such as macrophage has also been described, whereby metabolic signals such as exogenous heme or apoptotic bodies induce changes allowing the macrophage to respond to a later pathogenic and activating signal (25).

Leukocytes are recruited to the lung from the pulmonary and bronchial circulation, including alveolar capillaries and post-capillary venules. Recruitment of leukocytes to the lungs is a complex process, tightly regulated by both leukocytes and the vascular endothelium. In the bronchial circulation, or in larger vessels, the leukocyte trafficking cascade can be broadly split into three stages: rolling, firm adhesion, and extravasation or transmigration (26). Each of these stages has distinct groups of adhesion molecules that govern the interaction of leukocytes with the endothelium (27).

#### Rolling

Endothelial cells within the blood vessels detect chemokines and are able to rapidly increase the expression of P-selectin (CD62P) (28, 29) and E-selectin (CD62E) (30). These two selectins can be bound by P-selectin glycoprotein ligand-1 (PSGL-1) which, despite its name, can bind all three main selectins (CD62P, E and L) (31). PSGL-1 is expressed on the surface of neutrophils and monocytes (32, 33) allowing for increased interaction between activated endothelium and passing leukocytes (31). CD62L is the only selectin expressed by neutrophils (34), but also by monocytes (35) and is maintained on the plasma membrane (36). Human neutrophils are also able to directly bind CD62E with CD62L (37). PSGL-1 is also expressed by activated endothelium (38) and is, therefore, capable of also binding to CD62L on the surface of the leukocyte. Together, the expression of selectins and PSGL-1 results in rolling – a process that occurs under shear stress in the circulation, and indeed requires shear stress to function correctly (39). These interactions provide multiple points for pharmacological intervention to either block or enhance leukocyte recruitment to sites of activated endothelium.

#### Extravasation/Transmigration

At the point of firm adhesion and rolling arrest, two processes can occur: crawling along the vascular lumen or transmigration into the tissue. Intraluminal crawling has been visualised *in vivo* in mice using intravital microscopy, identifying the reliance on LFA-1 for initial adhesion and Mac-1 for efficient crawling (40). Transmigration predominantly occurs paracellularly (between endothelial cell junctions) (41), however, movement through the endothelial cytoplasm, known as transcellular migration (42), has also been described *in vitro* (43).

Two major signalling pathways have been identified as of central importance neutrophil chemotaxis: PI3K and MAPK (16). Responses to intermediate chemoattractants are heavily reliant on the dual action of phosphoinositide 3-kinase (PI3K), specifically the gamma and delta isoforms in human leukocytes, at the leading edge and phosphatase and tensin homolog (PTEN) at the lagging edge (44, 45) – two enzymes that control the phosphorylation of phosphatidylinositol. In contrast, p38 MAPK co-ordinates neutrophil chemotaxis to end-point chemoattractants (46).

The process of cellular recruitment through the pulmonary vasculature is thought to occur *via* slightly different processes, dependant on adhesion receptor expression (42). At their smallest diameter, tight and tortuous pulmonary capillaries have an internal diameter of less than 2µm, significantly smaller than a neutrophil, which, in an unpolarised form, has a diameter of approximately 7µm. Despite this, *in vivo* studies have demonstrated that in health, human neutrophils are able to pass through the pulmonary capillaries with a similar speed to red blood cells (47). Furthermore, the deformation of the neutrophil in passing through these capillaries may actually provide an innate mechanical mechanism to ‘de-prime’ neutrophils in the circulation: neutrophils that were artificially primed ex-vivo and reintroduced to the host circulation initially increased their transit time through the lungs, but this effect was slowly lost (47). Complementing these findings, forced mechanical deformation ex-vivo of neutrophils also reversed the changes observed in primed neutrophils, suggesting a mechanism of de-priming (48). Neutrophil transit though narrow capillaries, such as those in the pulmonary vasculature, might, therefore, have important functions for immunomodulation, allowing primed neutrophils to return to the quiescent state.

### Response Within the Parenchyma

Neutrophils are the first wave of leukocytes to arrive in the lungs upon infection, followed by monocytes (10, 49). To migrate through the dense and elastic extracellular matrix of the lungs, it has been suggested that neutrophils release small amounts of proteinases and then reactive oxygen species sequentially (50). Inflamed tissue tends to be hypoxic and lactataemic, conditions that promote neutrophil survival *via* a distinct signalling pathway involving hypoxia-inducible factor 1α (HIF-1) (51). In the lungs, neutrophils actively kill invading pathogens by a number of processes, including phagocytosis and by the release of antimicrobial molecules including reactive oxygen species (ROS) and neutrophil extracellular traps (NETs) (52). Once in the lungs and in response to inflammatory stimuli, monocytes differentiate into monocyte-derived macrophages (MDM) or monocyte-derived dendritic cells (MoDC) dependant on the microenvironment (53).

On resolution of inflammation, a proportion of neutrophils die by apoptosis, and many are cleared by macrophages through a process called efferocytosis (54). Apoptosis is triggered either by intrinsic loss of mitochondrial membrane integrity, causing release of cytochrome c into the cytoplasm and promoting activation of caspase 3; or by extrinsic signalling through death receptors to drive caspase 8-dependant activation of caspase 3 (55). Apoptosis triggers the externalisation of phosphatidylserine (PS), an ‘eat me’ signal, as well as downregulation of ‘don’t eat me’ signals CD47 and CD31. This process can be regulated by the cell, suggesting modulation of the pathways and receptors involved may be a mechanism by which efferocytosis of excessively trafficked neutrophils could be enhanced in lung disease (54).

Other mechanisms of clearance of neutrophils and other dying immune cells from the lung include *via* the mucociliary escalator (56), whereby ciliated epithelial cells covered with a mucus layer beat synchronously to move entrapped particulates, including cells, up to the throat for removal by expectoration. In lung diseases such as COPD and IPF, there are both increases in mucus production and expectoration and increases in the number of trafficking cells within these secretions (57, 58). Neutrophils may also leave the site of inflammation through a process known as reverse transmigration, whereby neutrophils migrate across the endothelium and re-enter the vasculature (59). Although not yet fully characterised this, and integrins which are needed for this activity, may be another therapeutic target.

Neutrophil phagocytosis occurs through direct interactions between bacteria and immune cells (“unopsonised phagocytosis”) but is more efficient when bacteria are coated with immunoglobulins and complement (“opsonised phagocytosis”). Optimal opsonisation requires both immunoglobulins and complement (60). However, unopposed neutrophil elastase can impede both immunoglobulin and complement activity, cleaving the hinge region of IgA and complement C3bi, forming a functional opsonin mismatch (61, 62) which may be important in predisposing the host to secondary lung infections in chronic illness.

Neutrophils are not the only cells implicated in these processes. Monocytes are able to phagocytosis to a small extent, but they are also key modulators of the immune response through inflammatory mediator release. In response to inflammatory stimuli, monocytes are induced to differentiate into MDM through high levels of GM-CSF in the lungs, which is elevated during inflammation (63). MDM add to the pool of local alveolar macrophages, and contribute to high levels of phagocytosis of bacteria, inflammatory mediator release and, on resolution of inflammation, efferocytosis of dying neutrophils and epithelial cells, to ensure safe clearance of these dying cells (10, 54). MoDC also supplement the local pool of dendritic cells, to take up infectious agents, process and present antigen on the cell surface, followed by migration to the lymph nodes to activate T cells and the adaptive immune response (64).

The containment of the inflammatory signal to where it is needed, for only as long as needed is especially important in lung tissue. The lungs rely on their elastic properties to maintain adequate ventilation. Elastic fibres are highly complex matrix structures because of their size, molecular complexity, and the requirement for numerous helper proteins to facilitate fibre assembly (65). Previous studies have conclusively shown that elastin degradation caused by neutrophil proteinases is a key step in the pathogenesis of many chronic lung diseases and that lung cells are unable to repair damaged elastic fibres, leading to permanently compromised lung function and ongoing degenerative disease (66).

## Changes With Age and in Lung Disease

Alterations in the innate immune response have been identified in lung diseases including COPD and IPF, but it is important to note that most lung diseases are more common with advancing age, and there are changes to both the structure and function of the lung and immune cells (including neutrophil) responses with age, which might influence cellular trafficking. This has identified a number of processes that could be targeted for treatment.

### Ageing

Increased age is associated with both elevated rates of infections and chronic lung disease, as well as worse outcomes after illness or injury. In the UK, 95% deaths from pneumonia (67) and 86% of deaths from influenzae (68) were in those over 65 years of age. During the COVID-19 pandemic, 73% of deaths recorded so far occurred in those aged 75 or over (69). Over 90% of those with COPD (70), 75% of those with IPF (71) and 90% of those with bronchiectasis (67) are aged over 65 years of age. This elevated risk is likely due to a number of factors. There are age-associated changes to the lung structure and function. These include a less compliant thoracic cage; a weaker diaphragm; less elastic lung parenchyma leading to senile emphysema; reduced efficiency of the muco-ciliary escalator reducing the clearance of bacteria and microparticles from the lung as well as a reduced ability to maintain homeostasis (including reduced responsiveness to hypoxia and hypercapnia) (72). Ageing is associated with chronic low grade inflammation characterised by increased basal levels of cytokines including Interleukin (IL)-1, IL-6 and tumour necrosis factor (TNF)-α (73). The function of the immune system can also alter with age, termed immunosenesence, with impaired innate and adaptive immune responses to infections and inflammation, and this includes alterations in most neutrophil cellular functions.

Neutrophils show a gradual decline in the accuracy of migration (chemotaxis) with increasing age, although chemokinesis, or the ability to move in any direction, appears unaltered (74, 75). Imprecise migration is thought to have significant consequences, leading to both a delay in reaching the site of inflammation, but also contributing to inflammation, as these cells appear to release both proteinases and reactive oxygen species during their convoluted migratory pathways. The deficit is associated with frailty, with adults displaying more pronounced frailty having the most impaired neutrophil responses (76). *In vitro* studies have suggested impaired migration can be restored to levels which reflect those of a younger adult by inhibiting PI3K, especially gamma and delta isoforms, indicating involvement of this pathway (77, 78). As well as alteration to migration, neutrophils that are recruited to the aged lungs show suboptimal superoxide generation and degranulation, and reduced phagocytosis (79, 80). The cause of these changes is unclear, but *in vitro* work suggests that merely exposing neutrophils to the inflammation found with age (by incubating cells from young adults with plasma or serum from older adults) is insufficient to reproduce the cellular phenotype, suggesting the altered functions are not merely a consequence of the inflammatory environment (76, 77).

Once again, these age-related changes are not only seen in neutrophils. Monocytes also show changes during ageing, with levels of intermediate (CD14++CD16+) and non-classical (CD14+CD16++) monocytes increased compared to younger adults. These cells also show impaired phagocytosis, altered cytokine release and elevated expression of migration marker CD11b (81, 82). On stimulation, aged monocytes produce less inflammatory cytokines including IL-1β, TNFα, IL-6 and IFNα (83–85) which may contribute to susceptibility to respiratory infection. As with neutrophils, there is a link with frailty and increased monocyte number, however it is as of yet unclear how this relates to cell function (86). Mitochondria in aged classical monocytes have reduced membrane potential compared to young monocytes, which may impair cell function due to impaired energy generation (87). Further analysis of metabolic effects of aging on monocyte function may reveal novel insights about the role of these cells in normal aging.

### Neutrophil Trafficking in Lung Disease

There is evidence that the detrimental changes to cellular function seen with age are heightened in lung disease and indeed most chronic and acute lung diseases are associated with an influx of neutrophils to the airways as well as neutrophilic inflammation. Hypoxia is another feature of lung disease, with the potential to alter neutrophil responses further, as described above. Indeed, across many lung diseases, pathogenesis and progression has been associated with an exaggerated and sustained presence of trafficking cells, with specific examples discussed below.

### Chronic Obstructive Pulmonary Disease

COPD is a common, debilitating and chronic disease thought to affect 10% of the adult population. It is currently the fourth leading cause of death globally and defined by persistent respiratory symptoms and airflow limitation which is associated with airways inflammation (88). The disease is often complicated by acute worsening of symptoms, termed exacerbations and commonly caused by viral or bacterial infections (89). Innate immune cells are considered to be key drivers of COPD. COPD is associated with greatly increased numbers of neutrophils in lung secretions, having been recruited from the systemic circulation into the airways due to epithelial damage, inflammation and infection (2). Both the lung tissue and secretions contain elevated numbers of macrophages (20x) as a result of elevated influx of monocytes which differentiate into monocyte derived macrophages (90–92).

Alveolar macrophages are likely to be a key driver of elevated leukocyte recruitment to the lungs during COPD, with COPD sputum and bronchi-alveolar lavage (BAL) containing elevated levels of CXCL-8 (93), LTB4, growth-related oncogene (GRO) alpha (94, 95), and MCP-1 (96), amongst other inflammatory agents.

Despite this high number of neutrophils and macrophages in the lungs or airways of patients with COPD, patients suffer with recurrent infections which suggests these cells are dysfunctional (53). In keeping with this, neutrophils from patients with COPD from mild to severe disease have been shown to have an increased speed of migration but also a reduced accuracy of migration towards single chemokines, bacterial products and sputum, associated with reduced pseudopod extension but correctable with PI3K inhibition (97). Of note, similar characteristics were seen in smokers aged between 30 and 40 years of age with respiratory symptoms including chronic bronchitis but no airflow obstruction (98), suggesting altered cellular functions are an early manifestation of disease. Once established the airways inflammation and altered cellular functions appear to persist even after smoking cessation, with heighted cell trafficking to the lungs seen even many years after the patients have stopped smoking (99).

Monocytes from COPD patients show enhanced migration to chemoattractant, which may contribute to enhanced levels of MDM in the COPD lung (100). These monocytes display a heightened pro-inflammatory phenotype, including elevated IL-6 and MCP-1 release (101), but do not show impaired phagocytosis compared to AMC (102). Monocyte-derived macrophage phagocytosis is impaired in COPD (102, 103), alongside impaired mitochondrial function (104) which implicates defective monocytes as pre-cursers to these cells.

### Idiopathic Pulmonary Fibrosis

Idiopathic pulmonary fibrosis (IPF) is a progressive condition believed to arise in genetically susceptible individuals as a consequence of an aberrant wound-healing response following repetitive alveolar injury. It is characterised by progressive deposition of extracellular matrix and collagen within the interstitium of the lung, leading to impaired gas exchange, breathlessness and eventually death. The involvement of leukocytes is acknowledged but remains unclear. CXCL-8 levels are elevated in IPF, with BAL neutrophilia a risk factor for early death. Neutrophil elastase damages epithelial cells, and it has been hypothesised that this damage and the subsequent release of DAMPS drives ECM component turnover. Indeed, NE deficient mice are resistant to bleomycin induced PF, however the role in humans is still unclear (105) just as it is unclear what drives the neutrophil recruitment to the lungs in the first instance.

Recently, a role for N-formyl peptide (fMLF) receptors (FPRs) has been described, which might be specific for lung fibrosis. FPR-1–deficient (fpr1–/–) mice are protected from bleomycin-induced pulmonary fibrosis but can develop renal and hepatic fibrosis as normal with the model utilised (106). It is known that infections can drive IPF progression, so potentially the neutrophils may have initially been recruited in response to an infective event, with subsequent recruitment reflecting the abnormal response to wound repair (107). However, the initiating driver of recruitment might reflect other stimuli, as an increasing number of alternative, non–formyl peptide ligands for FPR-1 are being uncovered. Monocytes and macrophages may play a role in disease pathogenesis, although this is yet to be fully determined. Depletion of murine LyC6 monocytes reduces both alveolar macrophages and fibrosis in mice (108), while in humans, an association has been described between monocyte numbers and survival in IPF, with a high monocyte count linked to poorer outcomes (109).

### Community Acquired Pneumonia

It is not just chronic disease where altered leukocyte functions, including neutrophil migration, are associated with poor outcomes. During Community Acquired Pneumonia (CAP), neutrophils are recruited to the airways in high numbers, with the alveolar spaces becoming filled with an exudate made up of inflammatory cytokines, immune cells and systemic proteins leading to hypoxia and ventilation/perfusion mismatch. Neutrophil functions have been shown to be impaired in CAP, with reduced migratory accuracy but increased degranulation and NETosis (110). Of note, in older adults, the defect appears sustained and to worsen with the severity of the infectious event, with less dysfunction in simple lower respiratory tract infections and most dysfunction when CAP is associated with sepsis (111). In this instance, dysfunction could be replicated by exposure to plasma from septic patients (110), suggesting the inflammatory systemic environment adds to the cellular dysfunction. This CAP neutrophil phenotype again appears correctable, with correction associated with improved patient outcomes (112), highlighting the potential benefit of targeting these cells.

## Current Strategies to Target Leukocyte Trafficking in Lung Diseases

**Figure 2:**
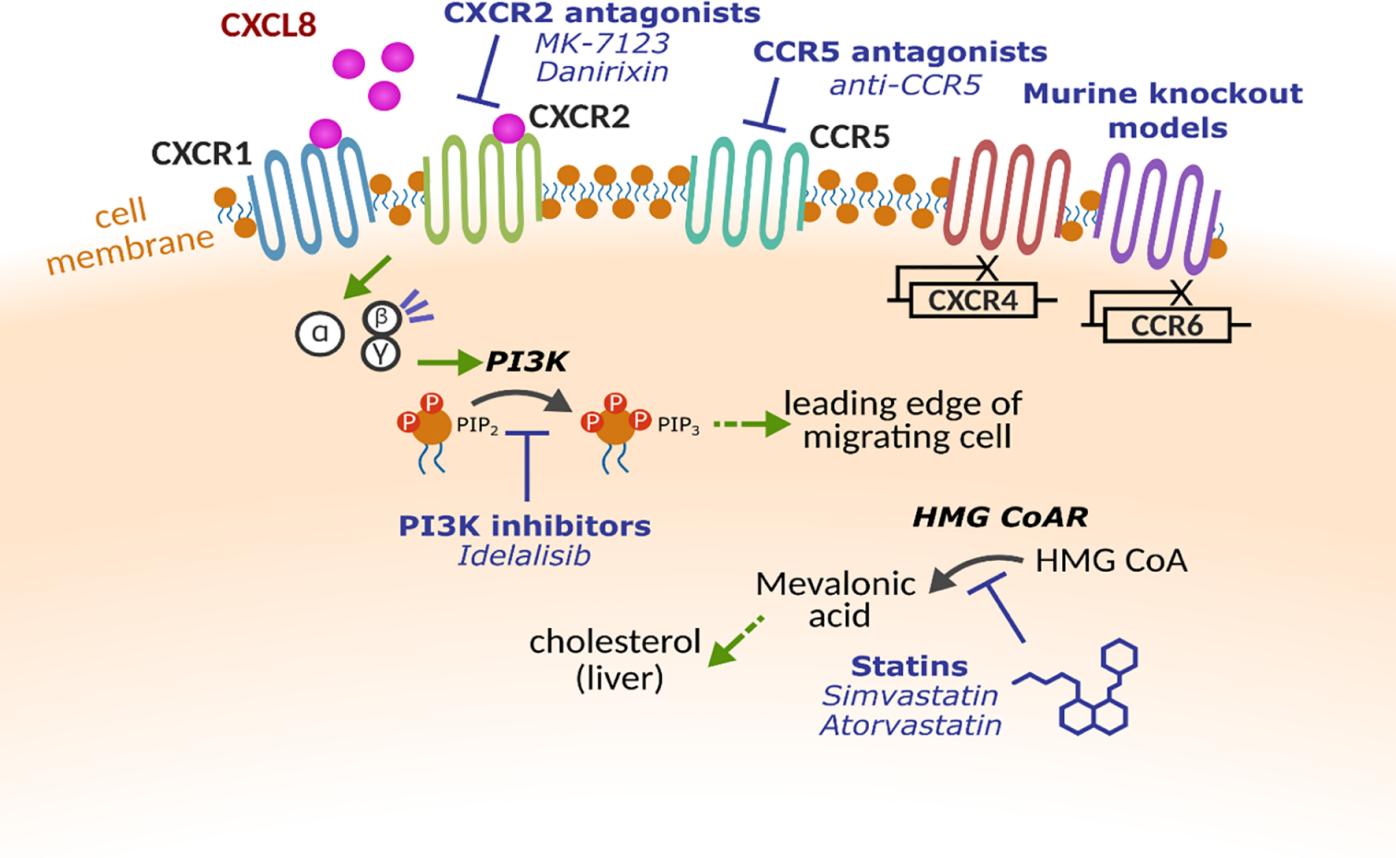
Molecular targets for altering leukocyte trafficking. Multiple receptors and proteins have been targeted to alter leukocyte trafficking. Chemokine receptors CXCR1, CXCR2, CXCR4 CCR5 and CCR6 have all been investigated either using pharmacological intervention or in early studies with gene knockout models. Within the cell, key enzymes such as phosphoinositide 3-kinase (PI3K) and β-Hydroxy β-methylglutaryl-CoA reductase (HMG CoAR) that have been implicated in cell motility.

The wealth of evidence describing the negative associations with lung disease and excessive or sustained leukocyte influx into the lungs highlights the need to target these processes, without compromising the hosts’ ability to respond to infections. A plethora of drugs targeting leukocyte trafficking have been developed, however, many to date have failed to make it to market for respiratory diseases. **Table 1** describes the potential targets and therapies developed, but key examples are provided below and **Figure 2** provides an overview of potential mechanisms.

**Table 1:** Therapeutic agents that target leukocyte function and their clinical trial results.

Category	Target (therapeutic agent)	Cohort	Outcome	Reference
**Priming agent**	TNF-α (Infliximab)	n=234 stable COPD, randomised	No therapeutic benefit	Rennard et al. (113)
	TNF-α (Etanercept)	n=81 AECOPD, randomised	No therapeutic benefit vs prednisone	Aaron et al. (114)
**Migratory stimuli**	LTB_4_ (BAYx1005)	n=17 stable COPD	Non-significant reduction in bronchial inflammation	Gompertz and Stockley (115)
**Migratory receptors (PMNs)**	CXCR2 (Danirixin)	N=614 symptomatic COPD, randomised	No therapeutic benefit, increased exacerbations in treated groups	Lazaar et al. (116)
**Proteinases**	Neutrophil elastase (Alvestat)	N=615 stable COPD, randomised	No clinical benefit	Kuna et al. (117)
	Alvestat	N=38 bronchiectasis, randomised	Improved FEV1	Stockley et al. (118)
**Migratory pathways**	PI3K (Idelalisib)	N=5 lymphoma/leukaemia patients	Impaired neutrophil functionality	Alflen et al. (119)
	Statins	N=62 CAP+S	Improved neutrophil chemotaxis	Sapey et al. (112)

A non-exhaustive list of the targets identified for leukocyte trafficking in lung disease, and the initial results of clinical studies.

### Targeting Priming Agents, Chemokines or Their Receptors

#### CXCR2 Inhibitors

CXCR2 is a major neutrophil and monocyte chemokine receptor, responsible for controlling migration towards ligands such as CXCL8. Inhibition of CXCR2 signalling is, therefore, an attractive target to dampen recruitment to CXCL8-rich tissue. The first report of a selective CXCR2 antagonist demonstrated reduced neutrophil migration to CXCL8 both *in vitro* using human neutrophils and *in vivo* blockade of neutrophil margination within rabbits (120). Several studies confirmed that blockade of CXCR2 reduced neutrophilic inflammation including in cigarette smoke-exposed rats (121); in an acute lung injury model in mice (122) and in an LPS airway challenge model in guinea pigs (123). Despite broad evidence from *in vivo* and *in vitro* models, clinical trials using CXCR2 antagonists provided a mixed picture.

The CXCR2 antagonist MK-7123 was used at various doses in a small phase 2 study including 616 patients with COPD, reporting that the highest dose of MK-7123 was able to improve FEV_1_ and increase the time to exacerbation, indicating a clinical benefit to patients. However, reductions in absolute neutrophil counts led to withdrawal of 18% of patients for safety reasons and there was also a significant increase in the inflammatory marker, C-Reactive protein (CRP) (124).

In a clinical trial of danirixin, another CXCR2 inhibitor, initial studies suggested clinical benefit in COPD (116). A subsequent larger trial (including 614 COPD patients) (116) found no significant clinical benefit in respiratory symptoms but significantly exacerbations and pneumonia events in the highest dose group, suggesting impairments in host responses to infection.

#### Targeting Other Chemokines and Their Receptors

LTB_4_ is a potent and proinflammatory chemoattractant, synthesised by neutrophils following the enzymatic conversion of arachidonic acid and facilitated by 5-lipoxygenase activating protein (FLAP). A study by Crooks et al. showed an increased concentration of LTB_4_ at presentation of infective exacerbation, compared to resolution of exacerbation, in a cohort of chronic bronchitis patients. Moreover, this finding coincided with an increase in sputum chemotactic and MPO activity, suggesting the role of LTB_4_ in bronchial inflammation (125).

Blockade of LTB_4_ was investigated in a phase II, randomised and placebo-controlled trial in a small cohort (n=17) of stable COPD patients (115). Participants were randomised to receive BAYx1005, an antagonist against FLAP, or placebo, for 14 days. Follow-up spontaneous sputum collection (day 14) revealed significant reduction of LTB_4_, compared to baseline, in the treated group. Although this reduction did not show complete suppression of LTB_4_, the observed change was similar to that observed at resolution of an exacerbation of chronic bronchitis. Hence, this trial suggested the efficacy of LTB_4_-blockade for the reduction of neutrophil-associated bronchial inflammation in patients with chronic lung disease.

Animal models of COPD have begun testing other chemokine receptor antagonists to identify promising candidates. *CCR6* and *CXCR3* knockout mice displayed reduced lung inflammation and evidence of protection against emphysema, when exposed to cigarette smoke (126, 127). Treatment with anti-CCR5 in an emphysema mouse model resulted in the reduction of apoptosis, DNA injury and alveolar remodelling with a subsequent reduction in lung inflammation (128). Whether there are any benefits of targeting these receptors in human disease remains to be tested.

#### Priming Agents

TNF-α is a priming agent for neutrophils, inducing their expression of β_2_ integrins and augmenting cell migration (129). TNF-α-induced degranulation, release of reactive oxygen intermediates and phagocytosis gives rise to local and systemic inflammatory responses (130). Given its pro-inflammatory consequences, studies suggest its role as a primary mediator of inflammation in COPD disease pathogenesis (131). Inhibition of TNF-α in COPD was investigated by Rennard and colleagues who conducted a randomised, placebo-controlled trial to assess the efficacy of TNF-α antagonism in moderate to severe COPD patients (113). A total of 157 patients were randomised to receive Infliximab, an anti-TNF- α antibody. No benefit was observed in the treated groups compared to placebo, in terms of changes in health status, lung function or exacerbation frequency. A later trial, using an alternative TNF-α-antagonist, sought to determine the efficacy of anti-TNF-α for the reduction of inflammation in a cohort of exacerbating COPD patients (114) with no clinical benefits.

### Targeting Intracellular Processes

#### PI3K

The PI3K pathway, activated by binding of ligands to G-protein coupled receptors, or tyrosine kinase receptors on the cell surface, is implicated in numerous leukocyte functions (132). Downstream effectors of PI3K activation include protein kinases that regulate cell motility and membrane trafficking, scaffolding proteins and other signalling processes (133). Neutrophils, monocytes, macrophages and T cells have all been show to require PI3K for chemotaxis, but also for phagocytosis through similar mechanisms of actin remodelling (134). *In vitro* experiments from neutrophils from older adults and COPD patients showed a relationship between inaccurate neutrophil migration and increased PI3K signalling, and that inhibition of PI3K*γ* or δ restored accuracy (77). Further *in vitro* experiments using idelalisib, a PI3K inhibitor used for non-Hodgkin lymphoma, showed that after TREM-1 ligation, idelalisib reduced L-selectin shedding, oxidative burst, degranulation and cytokine release in neutrophils (119). The reduction in all key neutrophil functions has led to some concerns about the potential safety of this therapy, with both the potential to normalise and neutralise neutrophil responses. In recognition of this, studies in COPD have used inhaled PI3K inhibitor therapies in the first instance, limiting systemic exposure. First reports suggest signals of clinical benefit (135), but wider trials are needed across all chronic lung diseases.

#### Repurposing Statins

Statins, or 3-hydroxy-3-methyl-glutaryl-coenzyme A (HMG CoA) Reductase Inhibitors are primarily used to treat dyslipidaemia. However, their association with reductions in all-cause mortality has led to further exploration of their anti-inflammatory and immunomodulatory properties. Several randomised controlled trials have demonstrated the reduction of systemic inflammation in cohorts treated with statins; a reduction in inflammatory biomarkers, namely high-sensitivity CRP (hsCRP) and IL-6, were observed in those treated with atorvastatin (136). Similarly, a downregulation of IL-8 and immune cell activation was seen in HIV patients treated with pitavastatin (137). Observational studies suggested that statins were associated with a reduction in mortality from pneumonia and influenza, despite patients taking these therapies being older and having more co-morbidities (138). Clinical trials have explored this further (139) and older patients with community acquired pneumonia and sepsis receiving 80mg simvastatin demonstrated improved neutrophil chemotaxis and reduced systemic neutrophil proteinase burden, as well as improving hospitalisation-free survival compared to placebo (140). Unfortunately, this benefit was not replicated in trials of statins in Acute Respiratory Distress Syndrome (ARDS) (for example (141),) leading to statins falling out of favour as an adjunct treatment. However, when patients were sub-stratified into those with the highest burden of inflammation, those with the most inflammation gained most benefit from a statin intervention (142), suggesting the need for careful patient selection.

In COPD the burden of inflammation rarely meets that seen in pneumonia or ARDS, however it was recently shown that neutrophils isolated from COPD patients, when incubated with simvastatin, improved their migratory dynamics towards CXCL8 and fMLP, to levels similar to aged matched healthy controls (143), indicating a potential benefit of statins directly on leukocyte migration. Meta-analyses have suggested that statins reduce not only cardiovascular risk in COPD, but also acute exacerbations and CRP (144) although this finding has not been universally (145), suggesting further studies are needed.

#### Targeting Neutrophil Proteinases

Neutrophil proteinases represent a promising target in chronic respiratory diseases, including COPD, AATD and IPF, as proteinases have been shown to be important in trafficking processes. There are a number of neutrophil elastase inhibitors under development (146).

In AATD, the clear association between neutrophil proteinases and lung disease has led to the use of augmentation therapy of infused AAT. This therapy is already licensed for use in some countries within Europe and the USA, but only for limited indications in the UK. Studies such as the RAPID trial (167 patients, placebo controlled) have demonstrated a reduction in the decline of lung function (147) and smaller studies have highlighted the positive impact of augmentation on neutrophilic inflammation (148). However, not all patients respond, and there is now interest in determining who gains the most benefit, for example by identifying and focusing on those with the fastest decline in lung function (149).

Alvestat (AZD9668) is a selective NE inhibitor with oral availability. In randomised control trials of COPD patients, 12 weeks of treatment with AZD9668 showed no positive effect on exacerbation frequency, symptoms, lung function or inflammatory biomarkers, but with 300 participants on active treatment, the study was likely underpowered for these heterogeneous outcome measures (117). Alvestat has also been studied in bronchiectasis, where 4 weeks of treatment improved FEV_1_, highlighting a potential signal of benefit (118). More recently, a trial of Brensocatib (an oral reversible inhibitor of dipeptidyl peptidase 1 (DPP-1), an enzyme responsible for the activation of neutrophil serine proteases) showed a reduction sputum neutrophil proteinases and improvements in clinical outcomes in bronchiectasis (150). This has renewed interest in anti-proteinase therapies, with many more trials in development or actively recruiting.

## The Challenges of Treatment Efficacy

Despite a strong rationale for targeting recruited immune cells, results of many trials have been negative. This might reflect the heterogeneity of the disease or population under study, a lack of stratification of the patient population, the wrong dose, modality or timing of the intervention.

Inflammation is very heterogeneous both within individuals and between individuals (95) and some studies may be underpowered to see changes in the biomarker they are assessing. Disease heterogeneity is also considerable. For example, COPD is an umbrella term for multiple pathologies and the resulting patient population can be very diverse. Attempting to treat all patients with the same therapy may hide the positive impact the treatment is having on some, due to a lack of effect in others. An example of both of these processes is that studies have highlighted a proportion of COPD patients with a polymorphism in the TNFα receptor, who experience an increased decline in FEV_1_, low body weight and altered sputum neutrophil recruitment which could be reduced with TNFα antibody (151). Potentially a lack of efficacy of TNFα in COPD trials might reflect a recruited population which has not been enriched for patients with this polymorphism. Other patient characteristics may also influence trial effectiveness. These include smoking status (as smoking retains the pro-inflammatory insult that triggered the disease initially), frequency of exacerbation, and the rate of lung function decline. The biology behind these differences in patient phenotype needs to be understood to allow new targets to be developed or repurposed therapies to be focused.

There have also been inconsistencies in the drug, dose and modality of therapies used in clinical trials. For example, the variable results from clinical trials of statins in chronic and acute lung disease might reflect differences in doses (with the greatest effects on cellular function seen at high dose), the population chosen (with most beneficial effects seen in older adults) and the timing of the intervention (with trials focusing earlier in the inflammatory journey having greater impact than those based within the Intensive Care Unit) (152). A more developed understanding will be needed across all these variables before the full impact of immunomodulatory targets can be harnessed for patient benefit.

## Conclusion

Leukocyte trafficking represents a promising target for the treatment of acute and chronic respiratory disease. These novel treatments could target the pathophysiology of disease, and so may provide significant impact for patients. However, often the complexity of immune cell trafficking and function and the heterogeneity of both patients and the respiratory disease have been poorly considered, with a “one size fits all” approach deployed in clinical trials. Our increased understanding of physiological and pathological immune cell responses provides an opportunity to rethink clinical trials in this space. Recent studies have shown more promise when targeting trafficking cells, and the learning from these studies have led to the expectation of a raft of new immunomodulatory therapies for lung disease in the near future.

## Author Contributions

KB, MH, DS, EW, and ES wrote the first version of the manuscript. ES finalised content. All authors contributed to the article and approved the submitted version.

## Funding

KB is funded by the MRC and Alpha 1 Foundation. MH is funded by The Wellcome Trust. EW is funded by the Alpha 1 Foundation. ES is funded by MRC, ISCF, EPSRC, ERDF, Alpha 1 Foundation.

## Conflict of Interest

The authors declare that the research was conducted in the absence of any commercial or financial relationships that could be construed as a potential conflict of interest.
